# An evaluation and comparison of shear bond strength of composite resin to dentin, using newer dentin bonding agents

**DOI:** 10.4103/0972-0707.44054

**Published:** 2008

**Authors:** Mithra N Hegde, Shruti Bhandary

**Affiliations:** Department of Conservative Dentistry and Endodontics, AB Shetty Memorial Institute of Dental Sciences, Deralakatte, Mangalore, India

**Keywords:** Dentin bonding agents, self etch adhesives, total etch adhesives

## Abstract

The purpose of this study was to assess the shear bond strength of Total etch Prime and Bond NT and self etch newer dentin bonding agents Clearfil S3, Xeno III Bond, Clearfil Protect Bond and G Bond used to bond composite resin to dentin, and to compare the difference in the shear bond strengths of the self etch newer dentin bonding agents. Hundred freshly extracted noncarious human maxillary premolar teeth were selected. The occlusal surfaces of each tooth were ground to prepare flat dentin surfaces at a depth of 1.5 mm and were randomly grouped, with twenty specimens in each: Group I - Prime and Bond NT, Group II - Clearfil Protect Bond, Group III - Xeno III Bond, Group IV - Clearfil S3 Bond, Group V - G Bond. Each group was treated with its respective bonding agents, as per the manufacturers' instructions Clearfill – Kuraray, Japan, G bond – GC Tokyo, Japan, Xeno- De Trey Densply, Germany. Blocks or Cylinders of composite resin were built up using Teflon mold and cured. Shear bond strengths were tested using Instron Universal testing machine and recorded in Mpa. The results were statistically analyzed using One-way anova and Tukeys HSD test. The total etch adhesive showed higher shear bond strength than self etching adhesives (*P* < 0.001). Within the limitations of this *in vitro* study, it can be concluded that all the adhesive agents evaluated showed optimal shear bond strength 17-20 Mpa, except G bond. However, shear bond strength of composite resin to dentin is better with one bottle total etch adhesive than with the newer self etching bonding agents.

## INTRODUCTION

Adhesive dentistry is a rapidly evolving discipline. For many years, the dental profession has strived to achieve good adhesion of resin composite to tooth substrate, since reliable bonding should produce less micro leakage and restoration stability.[1]

Way back in 1955, Buonocore introduced the concept of Acid etching, i.e. chemically treating the enamel to alter its surface characteristics to allow for adhesion of acrylic resins to the enamel surface of the tooth. Acid etching of the enamel gave way to total etch techniques, in which both the enamel and dentin surfaces are acid conditioned to allow for resin adherence to both enamel and dentin surfaces.[2]

In current times, development of new products is occurring at an unprecedented rate. Dentin adhesives are currently available as three-step, two- step, and single-step systems, depending on how the three cardinal steps of etching, priming and bonding to tooth substrate are accomplished.[3]

The newer concepts of self etching primers and adhesives have proven to be good both scientifically and clinically. They reduce the clinical steps, can be placed inexpensively, provide adequate bonding to enamel and dentin, and, most importantly, ensure post operative comfort for patients.[3]

The introduction of antibacterial properties into the bonding agents is another new concept.

The aim of this present study is to evaluate the shear bond strength of these newer dentin bonding agents.

## MATERIALS AND METHODS

**Figure 1:**
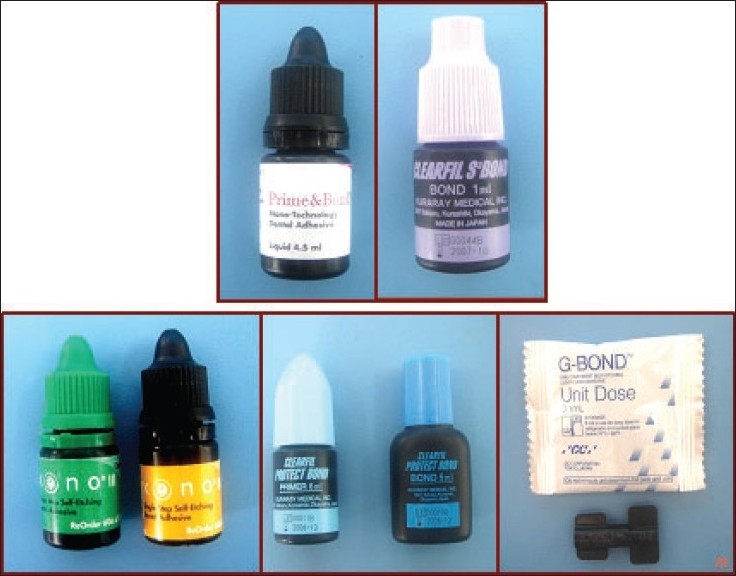
Bonding agents used for the study

Materials used in the study: [Figure 1]

## OPERATIVE PROCEDURE

### Preparation and grouping of the specimens for shear bond strength

Hundred recently extracted noncarious, intact, human maxillary premolars were selected. Teeth with restoration, cracks or other structural defects were excluded from the study. The occlusal surfaces of teeth were ground on water-cooled trimming wheel to prepare flat surfaces at a depth of 1.5 mm from the cuspal tip of the tooth.

They were randomly divided into five groups, with twenty specimens in each group, based on the dentin bonding agent used.

**Group I** - Prime and Bond NT (Control Group) Total etch Self-Priming
**Group II** - Clearfil S3 (Experimental group) One-step self etch.
**Group III** - Xeno III Bond (Experimental group) One-step self etch.
**Group IV** - Clearfil Protect Bond (Experimental group) Two-step self etch.
**Group V** - G Bond (Experimental group) One-step self etch.

Bonding agents were applied to all the specimens as per manufacturers' instructions Clearfill – Kuraray, Japan, G bond – GC Tokyo, Japan, Xeno- De Trey Densply, Germany.

**Table:** 

Bonding agent	Type	Composition
Prime & Bond NT	Total –etch	Di-& Trimethacrylate resins Functionalised amorphous silica, PENTA, Photoinitiators Stabilizers, Cetylamine hydrofluoride, Acetone
Etchant		37% phosphoric acid
Clearfil S3 Bond	One step-self etch	Bond: 10 MDP, Bis-GMA, HEMA, Hydrophobic dimethacrylate, Camphoroquinone, Ethyl alcohol, Water, Silanated colloidal silica.
Xeno III	One step-self etch	LiquidA: HEMA, Ethanol, Water, Highly dispersed silicon dioxide, BHT
		Liquid B: Phosphoric acid, Modified methacrylate, Monofluorophosphazene, Modified methacrylate, UDMA, BHT, Camphorquinone, Ethyl-4-imethyl aminobenzoate.
Clearfil Protect Bond	Two step-self etch	Primer:10-MDP,12 MDPB, HEMA, Hydrophilic dimethacrylates, Water Bond:10-MDP,Bis-GMA, HEMA, Hydrophobic dimethacrylate, Camphoroquinone, p-toluidine, Silanated colloidal silica, Sodium fluoride.
G Bond	One step-self etch	Bond: Acetone, 4-META, Water, UDMA, TEGDMA, Phosphate monomer, Fumed silica filler, Photoinitiators.

### Composite resin build-up

**Figure 2:**
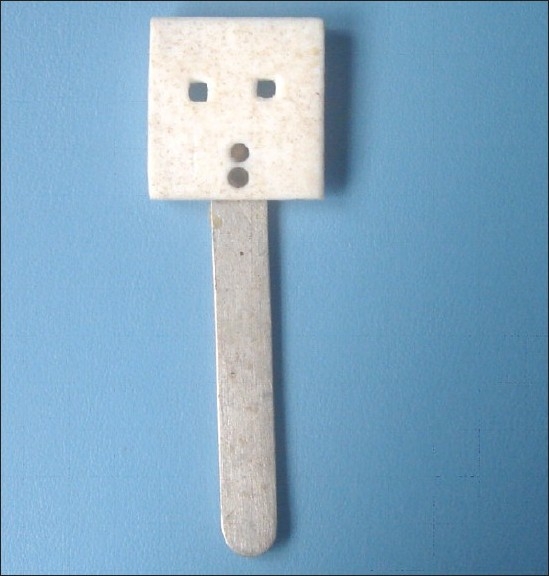
Teflon mold of dimension 2mm × 2mm

Filtek Z350 (3M) was then placed in increments, using a Teflon mold measuring 2 mm × 2 mm [Figure 2] and cured for 20 seconds on all the 100 specimens.

### Mounting of specimens

**Figure 3:**
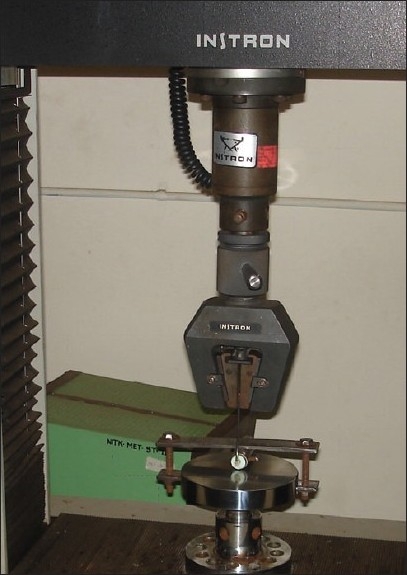
Instron machine for shear bond strength analysis

The prepared specimens were mounted on metal cylinders, using dental stone to embed the root portion. The study was conducted by placing the specimens in a distilled water bath for 24 hours, the temperature maintained at a controlled 37°C. All specimens were transferred to the Instron universal machine individually and subjected to shear bond strength analysis at crosshead speed of 1.0mm/minute [Figure 3].

## RESULTS

When a comparison of the shear bond strength of total etch with newer self etch adhesives was made using one-Way anova, it showed statistically significant results. [Table 1].

**Table 1:** Mean and standard deviation values for shear bond strength

Value	N	Mean	Std. deviation	Minimum	Maximum
Group I	20	26.092	.557	25.155	27.150
Group II	20	24.526	.534	23.555	25.545
Group III	20	24.858	.596	23.788	25.783
Group IV	20	22.060	1.148	20.050	23.785
Group V	20	16.378	.642	15.233	17.334

a. F=558.235 *P*<0.001 vhs

*P* < 0.001. Hence multigroup comparison was done using Tukeys HSD test.

The shear bond strength values of the control group (Group I), when compared with that of the experimental group (Group II, Group III, Group IV and Group V), showed statistically high significance. This indicated that total etch adhesives have better bonding capability than the newer self etching adhesives [Table 2].

**Table 2:** Intergroup comparison of shear bond strength values

Groups	Mean difference	t	*p*
Group I Group II	1.566	9.079	.001 vhs
Group I Group III	1.234	6.765	.001 vhs
Group I Group IV	4.032	14.131	.001 vhs
Group I Group V	9.715	51.119	.001 vhs

Group II Group III	-.333	-1.859	0.606 ns
Group II Group IV	2.466	8.707	.001 vhs
Group II Group V	8.148	43.639	.001 vhs

Group III Group IV	2.798	9.675	.001 vhs
Group III Group V	8.481	43.30	.001 vhs
Group IV Group V	5.683	19.32	.001 vhs

Intercomparison was done between the self etch adhesives using Tukeys HSD test. Comparison between Group II (Clearfil S3) and Group III (Xeno III) showed no statistically significant Figs. (*P* >0.05), which indicates that the two groups had comparable bond strength to dentin. Whereas, comparison of Group II with Group IV (Clearfil protect bond) and Group V (G bond) showed statistically significant results, leading to the inference that Clearfil S3 has a comparatively higher bond strength to dentin.

**Figure 4:**
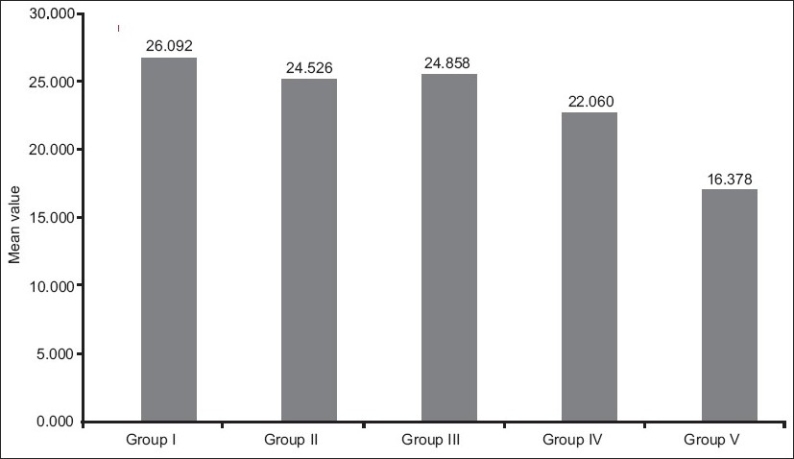
Bar graph showing comparison of the mean shear bond strength values between total etch (Group I) and self etch adhesives (Group II, Group III, Group IV, Group V)

When intergroup comparison was done between Group III and Groups IV and V using Tukeys HSD test, the results proved to be significant statistically, showing that Xeno III had better bond strength than Clearfil protect bond and G bond. Whereas, when Group IV and Group V were studied in comparison, the probability was < 0.001, which signifies that Clearfil protect bond has higher shear bond strength than G bond [Figure 4].

## DISCUSSION

It has been postulated that minimum bond strength of 17-20 Mpa is needed to resist contraction forces of resin composite materials, for enamel and dentin. Clinical experiences confirm that this bond strength is sufficient for successful retention of resin restoration.[4] All adhesive systems used in the present study achieved the optimal bond strength values for both enamel and dentin (except G Bond, which showed a slightly lower value). However, the total etch system Prime and Bond NT showed better bond strength, as compared to the self etching adhesives - Clearfil S3, Xeno III, Clearfil protect bond and G bond.

This result was in accordance with Bouillaguet *et al.*, Chuang *et al.*, Kerby *et al.*, who stated that self etching adhesives have lower bond strength as compared to total etch bonding systems.[5–7] Senawongse *et al.*, also demonstrated that two self etching systems, One-up bond and Clearfil SE bond demonstrated lower bond strength than the total etch system Single bond.[8] However, Kiremitci *et al.* concluded that self etching adhesive systems produced higher bond strength than conventional total etch systems, especially the all-in-one system, which produced the highest bond strength.[9] Whereas, Sensi *et al.*, stated that self etch and total etch primer showed comparable dentin bond strength.[9]

According to Hashimato *et al.*, self etch adhesives produced thinner and shorter resin tags than those produced by phosphoric acid etching and thus resulted in inferior bond strength as compared to total etch adhesive systems.[7]

Self etching adhesive systems rely on acidic monomers to simultaneously demineralize and infiltrate enamel and dentin. This acidity must be neutralized by the mineral content of the tooth structure, to allow complete polymerization of the adhesive film. With total etch adhesive, smear layer and dissolved mineral are removed during the rinsing step. Because of some questions about residual acidity and the fact that the smear layer is not removed, the issue of long term hydrolytic stability of the self etching adhesive systems still remains unresolved.[2]

Most single-step self etch adhesives contain hydroxyethyl methacrylate, which can polymerize in the presence of water to form microporous hydrogel with pore size ranging from 10-100nm. Differential water movement across the cured adhesive layer may occur in the presence of increased concentration of dissolved inorganic ions, uncured, water soluble, hydrophilic resin monomers or dissolved collagen proteoglycans components within the oxygen inhibition layer of the cured adhesive. This concentration difference may establish an osmotic pressure gradient, causing water movement from a region of low solute concentration to a region of high solute concentration. This may cause water blisters, which act as weak spots along the adhesive interface.[3]

Shear bond strength test is a simple evaluation procedure used to test the adhesion of dental adhesives Barkmeier and Cooley (1992). *In vitro* bond strength tests are useful and essential for predicting the performance of adhesive systems and possible correlation with clinical issues.[10] So shear bond strength testing is done with a universal testing machine, Instron, which is conventionally popular for evaluating the adhesive ability of adhesive/restorative materials. With the simple technique and relevant results, it is considered a benefit for the purposes of ranking and marketing.[7]

The lowest bond strength was obtained by the self etching HEMA-free adhesive, G bond. In a recent study, phase separation among adhesive compositions was confirmed, as droplets entrapped during solvent evaporation from HEMA-free adhesives. This phenomenon could be explained by the evaporation of solvents such as ethanol and acetone, which affected the balance of solvents and resin monomer and caused water to separate from other compositions of the adhesive.[11] Spherical blisters within the resin film may be the outcome of residual, free water, not completely evaporated and entrapped at the interfacial level. The convergence of small blisters into larger ones tends to produce honeycomb structures that may jeopardize the bonded interface.[12]

Clearfil protect bond, which is antibacterial two-step self etching adhesive, has a bond strength comparable with other self etch adhesives, even though it showed low bond strength when compared with Clearfil S3 and Xeno III. This is in accordance with study done by Imazato *et al.* in 1996, who found no decrease in bond strength by incorporating MDPB at any concentration. Imazato and Mc cabe (1994) demonstrated that a small improvement in the curing behavior of a Bis-GMA based resin was caused by incorporation of MDPB. It is well-known that the penetration of resin monomer into dentin surface and formation of a hybrid layer are important for resin dentin bonding (Nakabayashi *et al.*1982).

Achievement of strong micromechanical bonding depends on the depth of monomer penetration into demineralized dentin (Erikson 1992). It is possible that MDPB aids monomer penetration that generates good bond strength.[13] Clearfil S3 shows a comparatively higher bond strength among the self etching adhesives, but slightly lower than Xeno III (*P*>0.05). The reason attributed to this is the presence of MDP. This functional phosphate monomer determines its actual adhesive performance, to a large extent.

These self etch adhesives partially demineralize dentin, leaving hydroxyapatite partially attached to collagen. The residual hydroxyapatite chemically interacts with the functional monomer, determining the actual bonding efficiency and stability.[14]

According to a study done by sauro *et al.*, Clearfil S3 and G Bond showed reduced bond strength as compared to Clearfil protect bond, due to its increased permeability. Clearfil protect bond exhibited the lowest permeability and the fewest number of fluid droplets on the surface of the bonded surface. This inference is in contrast to the result obtained in this study in relation to Clearfil S3, because the simulated pulpal pressure effect was not experimented.[15]

In the present study, Xeno III, a one-step self etch adhesive demonstrated fairly good bond strength values with dentin. Van meerbeek *et al.* attributed the good bond strength values obtained with Xeno III to it being an intermediate strong self etch adhesive, with an acidic pH of 1.4. This acidic nature results in better micromechanical interlocking to enamel and dentin, as compared to mild self etch adhesives. It is also suggested that the residual hydroxyapatite at the hybrid layer base may still allow for chemical intermolecular interaction.[16]

Of all the adhesive systems tested, the total etch Prime and Bond NT showed the highest shear bond strength. The etch and rinse technique is still the most effective approach for achieving efficient and stable bonding and requires only two steps, primarily diffusion based, and depends on hybridization or infiltration of resin within the exposed collagen fibril scaffold, which should be as complete as possible.

Self etching adhesives are capable of penetrating the aqueous channels formed between the smear layer particles, widening these channels and interacting at the top of the underlying dentin. These agents offer a simpler clinical application than total etch systems, because they are capable of conditioning the tooth surface and simultaneously preparing it for adhesion. However, they provide lower bond strength than total etch systems because of their semi permeability, incorporation of smear layer, shorter resin tag formation, residual acidity and hydrolytic instability.

## CONCLUSION

Within the limitations of this *in vitro,* study it can be concluded that all the adhesive agents evaluated showed optimal shear bond strength of 17-20 Mpa, except G Bond. However, the one bottle total etch adhesive Prime and Bond NT recorded higher bond strength than the newer self etching bonding agents. In this study, it was seen that among the self etching adhesives, Xeno III showed the highest bond strength and G bond showed the lowest shear bond strength.
